# Bridging the gap in genetics: a progressive model for primary to specialist care

**DOI:** 10.1186/s12909-019-1622-y

**Published:** 2019-06-11

**Authors:** Brittany Harding, Colleen Webber, Lucia Rühland, Nancy Dalgarno, Christine Armour, Richard Birtwhistle, Glenn Brown, June C. Carroll, Michael Flavin, Susan P. Phillips, Jennifer J. MacKenzie

**Affiliations:** 1 Department of Pediatrics, McMaster University, Hamilton, Ontario Canada; 2 Queen’s University, 99 University Avenue, Kingston, Ontario K7L 3N6 Canada; 3 Botterell Hall, Queen’s University, 18 Stuart Street, Kingston, Ontario K7L 3N6 Canada; 4 Children’s Hospital of Eastern Ontario, 401 Smyth Road, Ottawa, Ontario K1H 8L1 Canada; 5 Centre for Studies in Primary Care, Queen’s University, 220 Bagot Street, P.O.#8888, Kingston, Ontario K7L 5E9 Canada; 6 Department of Family and Community Medicine, Granovsky Gluskin Family Medicine Centre, Mount Sinai Hospital, University of Toronto, 60 Murray St., 4th Floor, Box 25, Toronto, Ontario M5T 3L9 Canada; 7 Department of Pediatrics, Faculty of Health Sciences, Queen’s University, Kingston, Ontario Canada; 8 Department of Pediatrics, McMaster Children’s Hospital, 1280, Main St. West, 3N11-G, Hamilton, Ontario L8S 4K1 Canada; 9 Department of Public Health, Kingston, Ontario Canada; 10 Department of Medicine, Queen’s University, Kingston, Ontario Canada

**Keywords:** Primary care providers, Genetics, Genetic care, Continuing medical education, Undergraduate medical education, Prevention

## Abstract

**Background:**

The rapid expansion of genetic knowledge, and the implications for healthcare has resulted in an increased role for Primary Care Providers (PCPs) to incorporate genetics into their daily practice. The objective of this study was to explore the self-identified needs, including educational needs, of both urban and rural Primary Care Providers (PCPs) in order to provide genetic care to their patients.

**Methods:**

Using a qualitative grounded theory approach, ten key informant interviews, and one urban and two rural PCP focus groups (FGs) (*n* = 19) were conducted. All PCPs practiced in Southeastern Ontario. Data was analyzed using a constant comparative method and thematic design. The data reported here represent a subset of a larger study.

**Results:**

Participants reported that PCPs have a responsibility to ensure patients receive genetic care. However, specific roles and responsibilities for that care were poorly defined. PCPs identified a need for further education and resources to enable them to provide care for individuals with genetic conditions. Based on the findings, a progressive stepped model that bridges primary and specialty genetic care was developed; the model ranged from PCPs identifying patients with genetic conditions that they could manage alone, to patients who they could manage with informal or electronic consultation to those who clearly required specialist referral.

**Conclusions:**

PCPs identified a need to integrate genetics into primary care practice but they perceived barriers including a lack of knowledge and confidence, access to timely formal and informal consultation and clearly defined roles for themselves and specialists. To address gaps in PCP confidence in providing genetic care, interventions that are directed at accessible just-in-time support and consultation have the potential to empower PCPs to manage patients’ genetic conditions. Specific attention to content, timing, and accessibility of educational interventions is critical to address the needs of both urban and rural PCPs. A progressive framework for bridging primary to specialty care through a ‘stepped’ model for providing continuing medical education, and genetic care can was developed and can be used to guide future design and delivery of educational interventions and resources.

## Background

The rapid expansion of genetics in medicine has resulted in an increased role for Primary Care Providers (PCPs), which could include family physicians, nurse practitioners and nurses, to assess and educate patients about genetic risks and realities. In this current study, however, the PCPs are family physicians. No longer limited to rare conditions, genetics is increasingly important in the diagnosis and management of common conditions such as diabetes, hypertension, cancer, heart disease, and stroke; conditions that are leading causes of death in Canada [1]. With expanding clinical utility and demand for genetic care, including direct-to-consumer testing, PCPs [2–4] are increasingly called upon to integrate genetics into their practices [5–8]. Although there is a general increase in awareness about genetics, there remains concern that PCPs do not receive sufficient training in clinical genetics, that medical school genetic education curriculae do not address the practicalities of primary care practice, and that continuing medical education (CME) efforts have had a limited impact [9, 10]. The ability for PCPs to include genetics in their practice is especially relevant given the limited number of trained genetics professionals (Canadian Medical Association [Internet]), (Shuman, Personal Communications, August 9, 2017).

PCP perspectives of genetics have been explored over the past two decades in various countries and medical systems [3, 4, 8, 11–15]. Previous studies have demonstrated that PCPs perceived themselves to lack knowledge and understanding of genetics [11], and the confidence and resources required to implement genetic care into clinical practice [3, 5–8, 11–14, 16, 17]. Thus, genetics in clinical practice trails behind scientific and technological advances, which has the potential to impede management of patients and their families. Specific issues identified included a need for more knowledge about the modes of inheritance, environmental and genetic factors [5, 6, 18], the role of genetics in common disorders [15], and the type of information available [14]. PCPs indicated that they would benefit from more training in (i) linking family histories to risk assessments [4–7, 15, 16, 18], (ii) communicating and counselling patients about genetics (e.g. managing family dynamics and facilitating informed decision-making in a way that reduces fears and/or concerns, and helps guide patients through complex issues) [6, 14, 18–21], and (iii) knowing when and how to refer patients to a genetic specialist [3–5, 12, 16, 18]. Evidence suggests that PCPs would benefit from a better understanding about the options for early detection of disease, what genetic tests exist, how to interpret results, and prevention, management, and treatment strategies after a diagnosis [14, 15, 18]. For health care providers to be able to respond to the rapid increase in understanding about how genetic make-up shapes health and illness, medical education needs to transition from a traditional focus on the basic science of genetics to include a more clinical perspective [22–24]. PCPs suggested that this information is best taught through integrating genetics into existing pre-service medical education [14, 18] with CME used to educate current practitioners. Optimal CME programs would be accessible, short, engaging, timely, and either free or incentive-based [5, 14]. PCPs emphasized that education should incorporate a case-based practical approach, include information on clinical applications, focus on strategies to improve patient outcomes and practice (e.g., blended learning courses, online modules, PCP involvement in face-to-face or virtual genetics appointments), and be relevant to day-to-day clinical practice [4, 5, 7, 14, 25–30].

In spite of efforts to date, PCPs continue to express concern about inadequate knowledge, confidence, and resources, given the increasing role that genetics is expected to play in primary care [11]. Therefore, an up to date needs assessment of primary care providers is timely with a specific emphasis on genetic education needs. The objective of this study is to explore the self-identified genetic needs of PCPs, with specific consideration paid to the unique needs of both urban and rural PCPs. To date, few studies have explicitly considered the impact of practice locale on the availability of genetic education, whereas this study incorporates the perspectives of both urban and rural practitioners.

## Methods

A qualitative grounded theory approach was used [31]. Ten key informant interviews (*n* = 10) and three PCP focus groups (FGs) (*n* = 19) were conducted.The interview results were used to develop the FG protocol. The FGs explored PCPs perceptions of their current and future roles in providing genetic care, and the effectiveness of their genetic education, educational preferences, and perceived needs for improving future educational strategies. All interviews and FGs were audio-recorded and transcribed verbatim. Participants were de-identified to ensure confidentiality. All participants were given pseudonyms by assigning them a number for the key informant interview (I) or PCP focus group (FG). For example, informant number one was identified as (Informant 1), whereas, a PCP in FG one was identified as (FG1). The informants were also identified by their professional role (e.g., Informant 8GC refers to Informant Number 8 who is a genetic counsellor). NVivo 10 was used to store and manage the data. This study reports a subset of data from a larger study about genetics in primary care [11]. Ethics compliance was received from the Queen’s University Health Sciences and Affiliated Teaching Hospitals Research Ethics Board (File # 6005987).

### Key informant interviews

Stratified purposive sampling was used to recruit key informants to ensure a cross-section of perspectives and experiences. The key informants were selected by the research team and included one health care administrator (A), one clinical geneticist (CG), one nurse practitioner (NP), one public health medical doctor (PH/MD), two genetic counsellors (GC), and four primary care medical doctors (MD). Interview protocols were revised using an iterative approach as new themes emerged. All interviews were conducted by a distanced Research Assistant (RA) who had no prior involvement with the genetics program. To ensure authenticity of the data, member-checking was offered to all and completed by 4/10 participants. Each interview took approximately 45 min. Analysis of the interviews informed the development of the FG script.

### Focus groups

Participants for the three FGs were selected using purposive homogeneous group sampling to include only family physicians practicing in Southeastern Ontario (SEO). The Family Health Teams (FHT) in the region were contacted by the RA, and each local FHT administrator extended invitations to PCPs on their team and in the area. One FG consisted of urban PCPs (*n* = 5), while two FGs consisted of rural PCPs (*n* = 14). FGs were facilitated by a different distanced RA than the RA who held the interviews, and the Principal Investigator (PI) recorded field notes. The RA was selected for experience in facilitating FGs and had no prior involvement with genetics or with the PI. The FGs identified additional themes. Saturation of the data was reached with three focus groups. FGs took approximately one hour.

Both the semi-structured interview and FG protocols (Appendix 1 and 2) included open-ended questions that explored resource and educational needs, and preferred CME strategies for PCPs in SEO.

### Data analysis

Interviews and focus groups were analyzed through a grounded theory methodology, using the constant comparative method, to identify emerging patterns in the data [32–35]. Two RAs (LH, CW) used line-by-line open coding to independently analyze all transcripts. The RAs were selected for expertise in qualitative research analysis and had no experience in genetic research. The results were compared and revised until consensus about the emergent sub-themes and themes was reached. Using all data points, the codes were constantly compared to create eleven broader sub-themes of which four overarching themes emerged (see Table 1). The findings were then discussed with the research team until a final set of themes was created. Finally, the themes were used to provide the basis to develop a progressive model for bridging primary to specialty care. The trustworthiness and consistency of the data was ensured by using member checking, distanced RAs, analysis of the same scripts by multiple RAs, input of the research team, and correlation with the literature.

**Table 1:** Themes and sub-themes captured in interviews and focus groups

Theme	Sub-Themes
1. Roles and responsibilities of PCPs	Current demand exceeds supply of specialists; increased patient requests for education and testing
2. Genetic education needs	General knowledge
	Referral issues
	Managing patient care
3. Genetic education strategies to meet PCP needs	Medical education needs additional focus on clinical genetics
	Formal
	Informal
4. General considerations	Time constraints
	Awareness of educational opportunities
	Amount of knowledge necessary/appropriate for Primary Care Providers
	Rural-specific concerns

## Results

Ten key informant interviews (*n* = 10) and three PCP focus groups (FGs) (*n* = 19) were conducted, over 18 months. All participants worked in SEO. The PCPs in the interviews and FGs were between the ages of 30 and 60 years, and included men and women who had been in practice for a minimum of five years.

Participants acknowledged that PCPs have a responsibility to ensure patients receive genetic care. However, opinions were inconsistent about who would provide which type of genetic care. To ensure they provided appropriate care and referrals, PCPs believed that they required additional education. Participants highlighted educational strategies from which they would benefit and noted considerations that should be made when planning educational interventions. Table 1 identifies the four themes and 11 sub-themes that emerged from the data.

### Theme 1: roles and responsibilities of PCPs

Overall, most participants recognized that as the demand for genetic testing and care increases, this demand will exceed the supply of specialists. For example, one participant stated, “*I don’t know one single medical geneticist that is not overrun with work and I just can’t see how it’s going to get easier. I think it’s going to get much more difficult for them to facilitate their workload”* (Informant 1GC). Another participant suggested that PCPs’ key role lies in “*identification [of genetic conditions] and referral when appropriate, or [patient] education about the illness*” (Informant 7MD).

Another informant went on to explain that where cases do not warrant a referral to the specialist, PCPs must be prepared to offer,

*“the kind of psychosocial support that they [can] provide to patients in a very general way… in any circumstance [it] is relevant to the kind of support that one provides to individuals with, or at risk of, an inherited disease.”* (Informant 8CG)

### Theme 2: genetic education needs

All participants indicated that to appropriately refer and to provide genetic care, certain educational needs must be met. First, participants acknowledged the rapid growth of the field and application of genetics, and recognized that they require additional knowledge if they are to stay current:

*“You have to be pretty up to date on the evidence available to really do proper counselling and make recommendations especially if people are coming to you saying 'what should I do now’. Instead of giving them options, if you’re making specific recommendations then you need to be pretty comfortable with your level of knowledge and that it’s up to date.”* (FG3)

Most participants identified a need for education that would specifically guide decision-making processes about referrals. As one PCP stated, *“I would have to do some research on their [patients’] behalf to figure out what my role is next; if I refer to genetics, if I refer to a specialist, and if it’s necessary”* (FG1).

From the perspective of somebody who sees many of the PCP referrals, one genetic counsellor indicated that, “*There’s probably a lot of people that I’m not getting any referrals for, that could use the service, and part of it is that nobody knows it exists*” (Informant 3GC). Genetic counsellors acknowledged that making decisions about referrals was important for providing quality care: “*The biggest thing I think is recognizing that a patient may benefit, and then knowing where to refer or where to get additional guidance*” (Informant 1GC). The need for detailed information when PCPs referred patients to genetics was highlighted in addition to deciding which patients were appropriate to refer:

*“I think that’s the biggest thing, having more information when they send me the referrals... for a better ability to do triage…you can’t just send the referral form that says, please see this person.”* (Informant 3GC)

In acknowledging the value of genetic specialty expertise, one PCP stated:

*“I’ve been always very pleased when we refer someone for genetic counselling… and some expert spends time with the patient, gives them a ton of information and sends me a three-page letter at which I’m always amazed…. I know… [a] moderately small amount…. It would be nice to have something in the middle…. I sometimes think, this problem that [the patient is] concerned about is too small for the full genetic interview with a real expert, but I don’t know enough to give them something between the full consultation and my limited knowledge.”* (FG2)

When PCPs determined a referral was not necessary, most stated that they would benefit from increased education about how to provide genetic care within their practice. It was suggested that knowledge should be, “*enough… that you can appropriately counsel your patients*” (Informant 2MD), and include the “*positive and negative impacts the testing can have*” (Informant 10PH/MD).

### Theme 3: genetic education strategies

Participants identified a range of strategies and suggestions for education that they perceived as being particularly beneficial for them to enhance their ability to incorporate genetics into their practices. One participant states quite succinctly: “*The reality of any medicine is you’re always learning new stuff. And that would be the main thing I think for family docs is finding a way to keep up with specific knowledge based aspects of genetics”* (Informant 2MD).

Some participants suggested that undergraduate medical education should incorporate genetics into an array of subject areas, refrain from discussing it only in the context of rare conditions, and ensure consistency across medical schools. The educational challenge, however, continues to include strategies to enable health care providers to remain current in the rapidly evolving knowledge-base within genetics. Some participants suggested that undergraduate medical education provided adequate education but, due to the rapid growth in the field of genetics, knowledge obtained in medical school quickly becomes outdated: “*So I think in medical school and residency – the training we were provided with was up-to-date at that point. But it is not up-to-date any longer, depending on when they trained*” (Informant 10PH/MD).

Participants indicated that the continual need for genetics education could be accomplished both formally and informally. Formal educational strategies included continuing medical education (CME), lectures, seminars, or conferences—all with a focus on case-based learning. Participants discussed a range of CME options, with one saying, “*I personally like the CME format because it gets me there and it gets my full attention and there’s opportunity to interact”* (FG1). Others agreed that the “*most useful things are the good old fashioned face to face seminar[s]… the best way to educate family doctors is to come and give them re-education sessions... Come to the hospitals and the seminars and rounds*” (Informant 9PH/MD), or perhaps “*a half day conference just in genetics*” (Informant 7MD). Regardless of format, some participants suggested that, “c*ase-based learning actually would be the most helpful. Because… when you see a case and you learn about it, that’s when you remember it*” (FG3).

In addition to formal education sessions, participants also valued informal learning opportunities. PCPs reported that e-mail or telephone conversations with colleagues with genetic expertise could help educate them, and guide their decisions in practice: “*Having a person to bounce an idea off without having to make a formal referral that can be helpful*” (Informant 2MD). As well, multiple participants stated that mentorship from someone with expertise would be educationally beneficial. One participant recalled the value of hallway consultations with other physicians and suggested that facilitating this type of informal contact would contribute to improved genetic care, particularly in complex cases:

*“You’d have these hallway consultations with somebody … it was just a fantastic – he was the best resource. He was around most days and you’d run into him and you had this specific clinical scenario involving this individual patient. And you can’t really find the answer in books because this patient had a peculiar set of co-morbidities that made their answer really the only answer. And I think that whole idea of the ability to access some sensible expert to give you timely advice in a fairly, a user friendly way would be a superb tool. Not just for genetics but for everything–call this world medicine, e-curb site consult*.” (FG2)

Like this respondent, many participants discussed the importance of electronic resources. They found value in electronic resources that could be quickly accessed when searching for information in specific cases:

*“Websites where they can easily obtain the information and access what the next best steps should be.*” (Informant 5MD)

*“FAQ on genetics set up for family doctors… [with] answers to] basic questions, ‘Should I refer this person for breast cancer screening or whatever else? Yes or no.’ A little blurb on the evidence. And then a little thing down below saying – if you’re still having problems contact us. And you [could] fill your question and fire it off.*” (FG2)

Some participants discussed the benefits of an automated notification in the electronic medical record (EMR) which highlighted the need for genetic testing such that,

*“If you entered, ‘three people in the family who had breast cancer’ for example… not only [are you] entering the data in to figure out the risk... but inadvertently… you don’t even realize… that might be something that should trigger a genetics referral or another test… the EMR [asks] ‘have you considered ____ because you’ve entered three people with breast cancer in this person’s family history’… it prompts*.” (Informant 2MD)

### Theme 4: general considerations

With respect to formal and informal methods of education, participants asked that those responsible for planning educational interventions apply certain considerations. First, as one participant indicated, practitioners are not always “*aware of a lot of CME opportunities… [he is] not aware of a lot of outreach education”* (Informant 2MD), hence effective marketing strategies are key to increasing participation in genetic education offered to PCPs. Second, some participants drew attention to time constraints, pointing out that,

*“In this day and age, when everyone’s busy – it makes a lot of sense to do these webinars and all this kind of stuff. I find I don’t participate in them nearly as much because there’s always something else pulling your attention*.” (FG1)

Rural practitioners acknowledged specific challenges that distance could pose for receiving adequate genetics CME. One participant indicated that, “*there is easier access to in-services and education presentations at the urban centres as opposed to the rural hospitals or the rural centres*” (Informant 6A) while another stated that “*an [urban] physician that has access to ongoing rounds at a major university centre may have a different level of exposure to genetics*” (Informant 1GC). Finally, one participant reminded educators that the scope of PCP practice should be considered and that it should be acknowledged that PCPs cannot be expected to be genetic experts.

*“Remembering the details is not all that important as long as you remember that there is some aid. There is a piece of paper that [includes what] I need to know…about genetics that will give me some information about what to do with patients with genetic and sensitive issues. That’s the kind of superficial knowledge I think we need.”* (FG1)

## Discussion

**Fig. 1:**
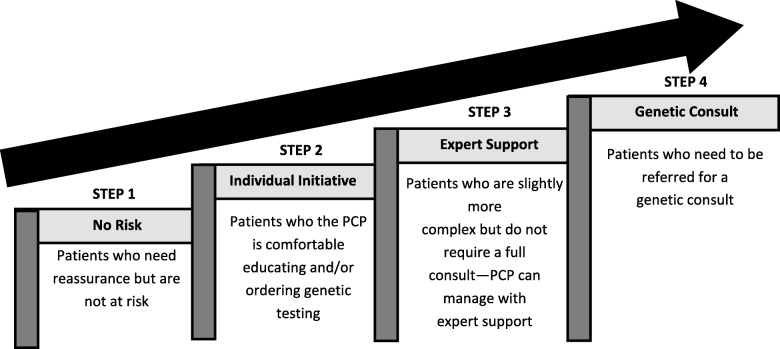
A progressive stepped model bridging primary and specialty care

The rapid expansion and increasing clinical utility of genetics in medicine requires that PCPs be prepared to provide genetic care for their patients. PCPs describe that their role is determined by patient needs and the complexity of the condition, as well as physician and logistical factors. With various aspects of genetic care being provided for by both PCPs and specialists, there is ambiguity surrounding the genetic care expectations and requirements of PCPs [15]. A framework for bridging primary and specialty genetic care through a progressive stepped model emerged from the data. Using this model, genetic care can be targeted to the patient and may range from providing education and reassurance, to performing genetic testing with or without specialist support, to referring patients for management by genetic specialists (Fig. 1). Step 1 includes reassuring the patients and requires minimal knowledge of genetics or confidence in discussing it by the PCP. Step 2 involves the individual PCP becoming comfortable educating and/or ordering genetic testing for a patient. This requires an increased level of knowledge about genetic care and the various tests possible. In Step 3, the PCP requires educational interventions that allow them to become effective in managing more complex patient cases that do not require a full consult. However, managing these patients does require PCPs to seek support from an expert. The final step, describes PCPs who are knowledgeable and confident enough about genetics to realize when a patient should be referred for a full genetics consult. This stepped model provides a method for PCPs to be more involved in the genetic care of their patients and clearly define when collaboration is required with a genetics specialist.

The advantage of this model is that it provides a developmental and sequential continuum of primary to tertiary care for increasingly complex cases. It also provides a framework to make the roles of different care providers more explicit. PCPs view their role as providing the care they feel capable of and determining which patients warrant a more detailed assessment. However, PCPs have often received little formal education in genetic medicine and have had limited exposure to the identification and management of patients with genetic conditions. Therefore, while PCPs feel a responsibility to provide genetic care to their patients, many discussed a lack of knowledge, confidence, and resources to do so and, therefore, are either not comfortable or limit the degree to which they integrate genetics into their daily clinical practice. In spite of efforts at CME, these findings are consistent with prior literature [3–6, 12, 14, 16, 18, 20, 26].

Many PCPs expressed a concern that they were unsure when referral was appropriate, and expressed a need for education or a guide which can be used to help support decision-making surrounding referrals to genetics. PCPs want a contact they can e-mail or call to informally discuss a case to determine whether to refer or manage a patient themselves. We recommend a more explicit and accessible role for e-mail and e-consults as a corridor consultation method given that systems change is needed if PCPs are going to play a larger role in genetic care. For example, an approach to this e-consult model could be in the form of a single email address to which PCPs can direct their questions. Once the email is received by a centralized location, it is forwarded to an on-call genetics specialist who would respond within a specified timeframe. The specialist would assist the PCP in determining which step(s) would be most appropriate be it to assist in reassuring the patient, provide education, offer expert advice about the specific tests needed, or expedite a referral, as described in our ‘stepped model’. An electronic forum would also address some of the barriers outlined by rural PCPs. The challenge in implementing this approach to genetic care lies in PCP access to genetics colleagues/experts in a timely manner. In addition, in some jurisdictions, access to funding for genetic tests requires a geneticist consult.

To improve PCP confidence in meeting patients’ genetics needs, participants made specific recommendations. PCPs’ suggestion of improving undergraduate medical education in genetics through an integration across disciplines is consistent with other reports [14, 18]. Incorporation of genetics into Family Medicine residency programs was also considered important. Consistent with CME literature in general, PCPs discussed the need to improve formal CME opportunities and to utilize a case-based approach that connects theory to practice [14, 25]. In addition, participants also emphasized the value of informal learning that occurs through regular contact with other professionals, with a goal to avoid unnecessary referrals. For example, “just-in-time” consultations with experts or methods wherein a librarian is employed to help PCPs locate necessary diagnostic information, have been shown to improve the speed and quality of PCP decision making, subsequently having the potential to improve patient access to care [36].

Regardless of method, we found that participants highlight that when considering education specifically intended for PCPs, program developers and organizers must consider time constraints, the level of the learner, and understand that PCPs cannot be expected to be as knowledgeable as genetic experts [7], thus set realistic limits on what PCPs are expected to know. Timely access to information would also assist PCPs in effectively embedding genetic care into their practices [5]. Education should be relevant to daily practice and include information on clinical application [5, 14, 26]. These PCP-focused educational aspects are supported in the literature, however, our results suggest that PCPs must be made explicitly aware of educational opportunities. Improved access to genetic education would allow other healthcare providers, such as nurses and nurse practitioners, to participate in assessing genetic risk factors that may include taking family histories and providing patients with education around their concerns.

### Considerations for education of rural practitioners

Rural PCPs identify some specific concerns regarding access to education. Most importantly, they highlight that distance is a significant barrier to both formal and informal means of education [14]. Lack of regular contact with larger academic centres, leads to fewer informal learning opportunities for rural PCPs compared to their urban counterparts [37, 38]. As programs are developed it is essential to include options (such as just-in-time e-mail access to an expert) that increase CME accessibility and informal interactions regardless of geographic location.

### Limitations of study

This study was conducted in SE Ontario with a small number of PCPs and is, therefore, limited in terms of generalizability at a national and international level. However, we do believe that the progressive stepped model for bridging primary to specialty care can be adapted to specific contexts within a healthcare system and inform future CME interventions.

## Conclusions

In summary, while PCPs identify a need to include genetics in their practices, they perceive that a lack of knowledge and resources is one of the major factors that impedes their ability to provide quality patient care in genetics. To address gaps in PCP knowledge, a diverse set of educational opportunities and interventions should be made available in order to meet the varying needs of different PCPs and include specific attention to content, timing, and accessibility.

The findings from this research are pertinent to the development of future educational opportunities and interventions. While educational interventions aimed at improving PCPs’ knowledge of genetic medicine may increase knowledge and perceived competence in genetic medicine, they are not always accompanied by a change in practice or referral patterns [39–42]. The progressive stepped model of bridging primary and specialty care developed from this study has the potential to assist in bridging the gap between primary care and genetics expert settings. Supporting PCPs in the care of patients they are uncertain about can be addressed with just-in-time strategies including timely access to experts. With the current study and the previous literature in mind, future research should not only assess the educational merit of opportunities and interventions, but should also seek to assess to what extent, and in which situations PCPs support the integration of genetics into their practices. To optimize the future of genetic care, ongoing support is needed to facilitate greater informal as well as formal collaboration, including the sharing of knowledge and skills between PCPs and genetic specialists.

## Data Availability

The datasets used and/or analysed during the current study are available from the corresponding author on reasonable request.

## Appendix 1

### Interview Protocol

1. When you think of genetics in primary care what do you think of?
2. Have you treated patients with genetics concerns or illness?
3. How often would you say you have patients in your practice with genetic issues?
  i. Would you typically mange the patient, once the genetic diagnosis is made or would you refer them on to another physician to do this?
4. How often do you order genetic tests? And what tests would you actually order?
5. Do you know the laboratories that do the genetic testing for the tests you order? And doyou know how they’re paid for it?
6. Do you know what requisition you use to order the test?
7. What cases would you say are referred or not?
8. What would you say is the role of the primary care providers with regard to genetics?
9. Do you think that primary care providers should provide more or less genetic testing?
10. Would you say that the role of primary care providers would be different in a rural vs urban setting?
11. And what do you think the family physicians needs to be aware of with respect to genetic issues in their patients?
12. Do you think genetics is important in primary care now?
13. What about in the future? How do you think genetics will be important in primary care in the future?
14. How will primary care evolve in response to genetic discoveries?
15. What skills do you think that primary care providers might need to meet these evolving practice needs?
16. Do you think that there are genetic resources that would help to family physicians, like diagnostic testing or counselling resources?
17. Do you that leaders in your region are keeping you up-to-date?
18. Is there genetic diagnostic, testing, or counselling resources that you think would be helpful to family physicians?
19. Are there genetic tools that you think would be helpful for family physicians?
20. Do you have any suggestions for education for primary care providers?
21. Is there anything else that you’d like to mention?

## Appendix 2

### Focus Group Protocol

1. There is broad coverage of genetics in the popular press and medical journals. It seems genetics is having an increasingly important role in medicine.

i. When you think about genetics in your daily practice what do you think of?
ii. What role does genetics play in your practice?
iii. Can you describe some experiences from your practice?

2. How do you view your role in the genetic care of a patient?

i. What are your thoughts about the purpose of genetic testing? In what situations would it be useful?
ii. Do physicians have a gate-keeper role to play with respect to genetic counselling/testing?
iii. Is this an area of interest to you?

3. Consensus among key informant interviews was that genetics will play an increasing role in primary care practice. There were also concerns about barriers to genetic care in practice. What barriers do you foresee?

i. What ideas do you have to address these barriers?
ii. Are there potential problems that may arise with patients being more aware of genetics and personalized genetic testing?

4. What competencies should family physicians have in genetic care?
5. What would facilitate the incorporation of genetics into primary care?

i. In your experience who has provided genetics educational support and resources for your practice?
ii. Who do you think should be providing this support?
iii. What strategies/solutions have you used to increase integration of genetics into your practice?
iv. What has been the most successful? What has been less successful?
v. Which resources are the most useful?
vi. Do you have a preference for:

- CME?
- Information sessions?
- Emails?
- Webpage?

vii. Which method have you found to be the most useful?
viii. Which would you like more access to?
ix. What screening tools would helpful – cancer? Cardiac? EMR/Hard Copy?
x. What is your perspective on using an EMR based genetic screening tool?

6. One of our goals is to understand the unique challenges of incorporating genetics into PCP in a rural setting. How would you describe these challenges?

i. How are the challenges in a rural practice different compared to an urban setting?
ii. Does an urban setting also entail unique challenges?
iii. Are the priorities different in a rural setting? For patients? For physicians?
iv. If there is a proportionately low referral rate from rural areas– what are the potential causes? If services aren’t available does that mean no testing?
v. What strategies or resources could be successful to integrate genetics into rural care? (e.g., Different models - just ordering tests, curb side consultations)

7. There are ongoing advances in the understanding of the uses of genetics in medicine. How do you anticipate that your use of genetics in practice will change over time?

i. Do you foresee family physicians having a greater role in genetic counselling and testing?
ii. Is there a value in genetic counselling in primary care?
iii. What impact do you anticipate that the integration of genetics into primary care will have on health resources?
iv. How will family physicians manage the increasing number of conditions that can be tested for and potentially treated?

8. Could you share any additional information that you think it would be helpful for us to be aware of?

## Abbreviations

A: Health care administrator
CG: Clinical geneticist
CME: Continuing medical education
EMR: Electronic medical record
FG: Focus group
GC: Genetic counsellor
MD: Medical doctor
NP: Nurse practitioner
PCP: Primary care provider
PH/MD: Public health medical doctor
PI: Principal investigator
RA: Research assistant
SEO: Southeastern ontario
